# Cl II Malocclusion Treatment, Using the Modified Twin Block Appliance Coordinated with Fixed Orthodontics in a Postmenarche Patient

**DOI:** 10.1155/2017/2525374

**Published:** 2017-04-12

**Authors:** Amin Aminian, Shahriar Sarvareh Azimzadeh, Elina Rahmanian

**Affiliations:** Kerman Oral and Dental Disease Research Center, Kerman University of Medical Sciences, Kerman, Iran

## Abstract

Functional appliances have been used for treatment of Class II patients for a long time. The main objective of therapy with functional appliances is to induce supplementary lengthening of the mandible by stimulating increased growth at the condylar cartilage. The Twin Block appliance is one of the most commonly used functional appliances. The aim of this paper is to present a case report of mandibular deficiency treatment with Twin Block appliance in a female patient whose sexual maturation (one and a half years after menarche) and cervical vertebral maturation stage indicated the end of the growth peak. The treatment started with bonding 0.022 in MBT prescription brackets on the upper arch in order to align upper teeth and create a symmetric overjet. When reaching alignment, a modified Twin Block was given to the patient for 8 months. Final coordination was achieved with fixed appliances in both arches. At the end of the treatment, profile of the patient improved, crowding was relieved, and Cl I relationship with normal overjet and overbite was achieved.

## 1. Introduction

Functional appliances have been used for over a century in the treatment of Class II patients. These appliances have been used in clinical orthodontics for a long time [1]. Functional appliances can be removable or fixed [2]. The Twin Block appliance is one of the most commonly used functional appliances, partly due to its acceptability by patients [3]. The main objective of therapy with functional appliances such as Twin Block is to induce supplementary lengthening of the mandible by stimulating increased growth at the condylar cartilage [2]. It has been demonstrated that the effectiveness of the functional treatment of mandibular growth deficiencies strongly depends on the biological responsiveness of the condylar cartilage, which in turn is associated with the growth rate of the mandible [4].

The rate of mandibular growth, however, is not constant throughout the juvenile and adolescent periods, with the existence of a pubertal peak in mandibular growth described in classical cephalometric studies [5–10]. The onset, duration, and intensity of this pubertal spurt in mandibular growth vary on an individual basis. Most of the literatures mentioned that one of the main indications of skeletal maturity marking the end of growth in females is menarche [4]. Another more recent indication of growth is cervical vertebral maturation stage (CVMS). With regard to this method, the peak in mandibular growth occurs between CVMS III and CVMS IV. CVMS V is recorded at least one year after the peak, although these indications might be affected by individual's variations from norm [11].

The following is a case report of mandibular deficiency treatment in a female patient whose sexual maturation (one and a half year after menarche) and CVMS (CVMS V) indicated the end of the mandibular growth.

## 2. Case Report

A 13-year-old female in her permanent dentition is presented with the chief complaint of crowded anterior teeth and retruded lower jaw. As mentioned before, there were signs that growth spurt was completed. The patient had a chin deficiency with decreased anterior facial height. There was no facial asymmetry and the lips were competent with deep mentolabial fold.

**Figure 1:**
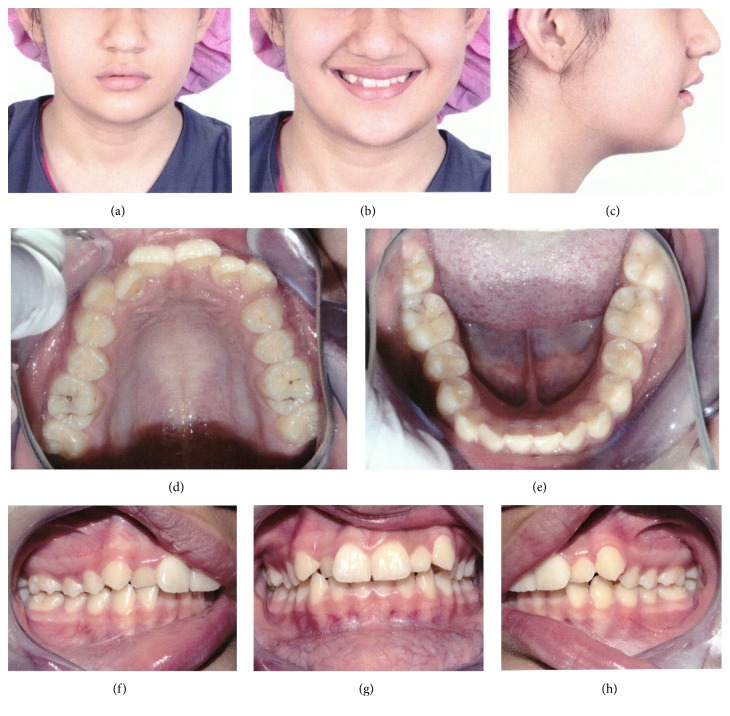
Initial photos: frontal (a); smile frontal (b); profile (c); intraoral views: upper occlusal (d), lower occlusal (e), right (f), frontal (g), and left (h).

In the intraoral assessment, the oral hygiene was fair but needed improvement prior to orthodontic treatment. In addition, some caries were detected that had been taken care of before starting the orthodontic treatment. Dental midlines in both arches were coincident and also coincided with the facial midline. There was a mild crowding in maxillary arch together with a mild lower anterior crowding. Angle classification was Class II division 1, and the buccal segment relationship was one-unit Class II on both sides. The overjet was 6 mm, whereas the overbite was 80% (Figure 1).

**Figure 2:**
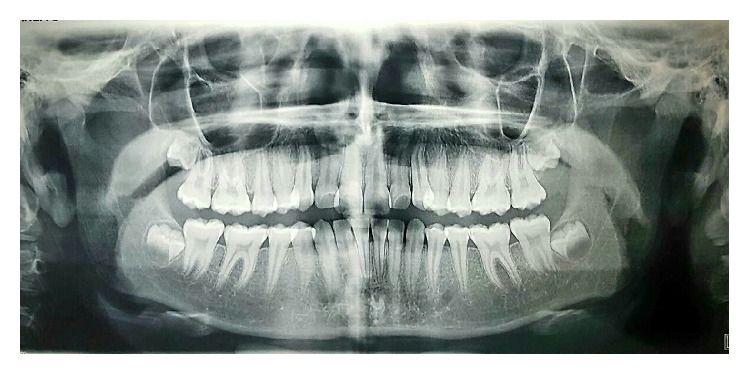
Pretreatment panoramic radiograph: the presence of all permanent teeth including the developing third molars.

**Figure 3:**
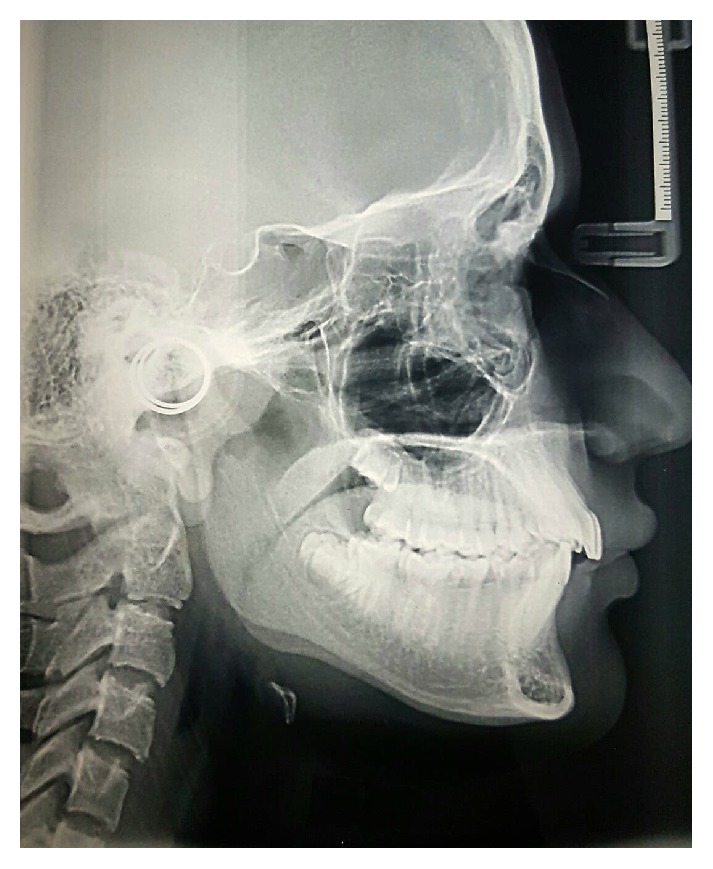
Pretreatment lateral cephalometric image.

The panoramic radiograph confirmed the presence of all permanent teeth including the developing third molars (Figure 2). In the cephalometric assessment (Figure 3), the ANB value of 4.2° suggested a mild Class II skeletal pattern. SN to mandibular plane angle had decreased. The upper incisors were proclined (U1-SN 112 degrees) and the lower incisors were of average inclination (IMPA 91.9 degrees) (Table 1).

Possible treatments are as follows.

The upper right and left posterior segments could be distalized using cervical headgear appliance to correct the molar relationship and create space to reduce the overjet and the overbite. Even though the headgear treatment has a restraining effect on the maxilla [13] it has little effect on mandible [11]. We can also use functional appliances, fixed or removable, which have more effect on mandible and also have a good control on both upper and lower dental arches. However both treatments rely on patient's cooperation and age.

It could be argued that this case might be treated only with fixed appliances using Class II intermaxillary traction; nevertheless, the main disadvantages with this approach are that it will lead to proclination of the lower incisors and molar correction to Class I may be difficult to achieve and has no skeletal effect. Besides, premolar extraction would be a possible treatment but has minimum effect on retruded mandible and may lead to increased overbite.

The patient was highly motivated and was also aware of possible treatments due to her personal researches. She asked for a complete treatment and also wanted her profile to be improved.

After thorough clinical examination, checking out the documents, and considering patient's motivation and expectations, we came up with the following phases of treatment.

The main objectives for phase I of the treatment (using fixed appliance for upper arch) were as follows:

1. Aligning maxillary arch
2. Creating a symmetric overjet for Twin Block therapy

As for phase II of the treatment (Twin Block therapy), the main objectives were as follows:

1. Reducing the overjet and the overbite
2. Improving the profile
3. Achieving Class I molar relationship

And in phase III the aims were as follows:

1. Relieving the lower arch crowding
2. Leveling, aligning, and coordinating the arches

**Figure 4:**
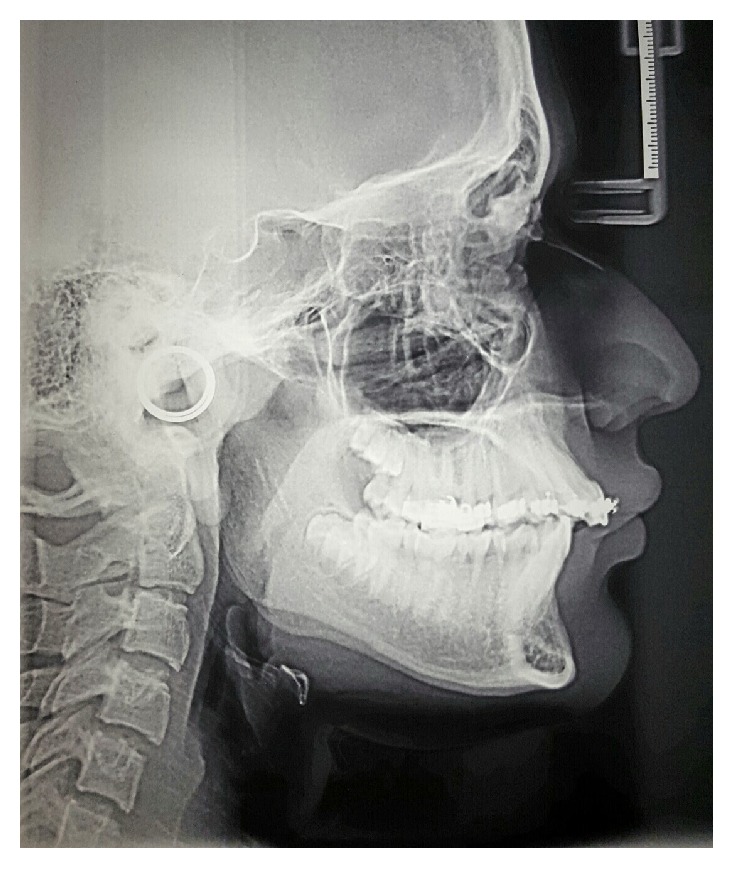
Cephalometric image after phase I (before starting Twin Block therapy): increased U1-SN angle and overjet are evident.

**Figure 5:**
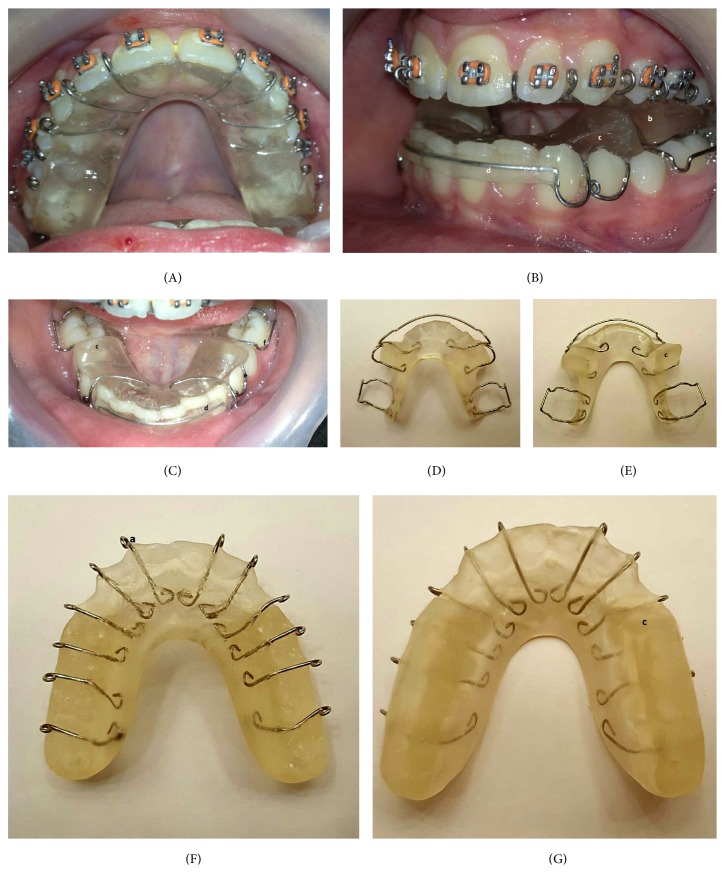
Twin Block appliance: maxillary part (A): (a) modified ball clasps used instead of conventional clasps to coordinate with fixed appliance; Twin Block appliance (B): (b) block A, (c) block B, (d) labial bow, (e) C clasps, and (f) Adam's clasp; mandibular part (C); mandibular part mucosal surface (D); mandibular part occlusal surface (E); maxillary part mucosal surface (F); maxillary part occlusal surface (G).

**Figure 6:**
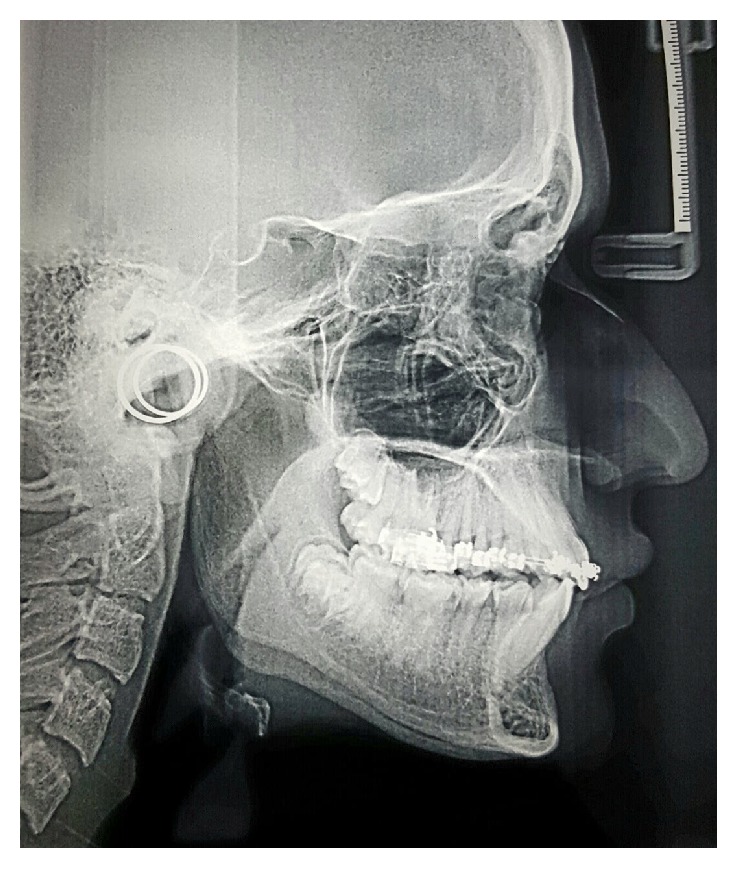
Cephalometric image after phase II (discontinuing Twin Block): mandibular growth is evident.

In the first phase of the treatment we bonded 0.022 in MBT prescription brackets on the upper arch in order to align upper teeth. Alignment was achieved with the use of NiTi wires, followed by stainless steel (SS) wires. Three months after the alignment phase has started, we took impression for Twin Block appliance while 0.016-inch SS wire was in the brackets (Figure 4). After that, a Twin Block that could be used with fixed appliance was given to the patient (Figure 5). The patient was then asked to wear the appliance full time except for mealtime and tooth brushing. It took 8 months to achieve the molar relationship overcorrection, that is, zero overjet without any apparent anterior occlusal shift. Since then, the patient was asked to use the appliance at night for another 2 months as retention. At this stage lateral cephalometric image clearly showed mandibular growth (Figure 6). The third phase of the treatment was performed by discontinuing the appliance and bonding the lower arch. In this phase, we did not use any other Class II correction mechanics such as Class II correcting elastics. Retention was achieved with Hawley appliance in maxillary arch and a fixed spiral wire in mandible, from canine to canine.

**Figure 7:**
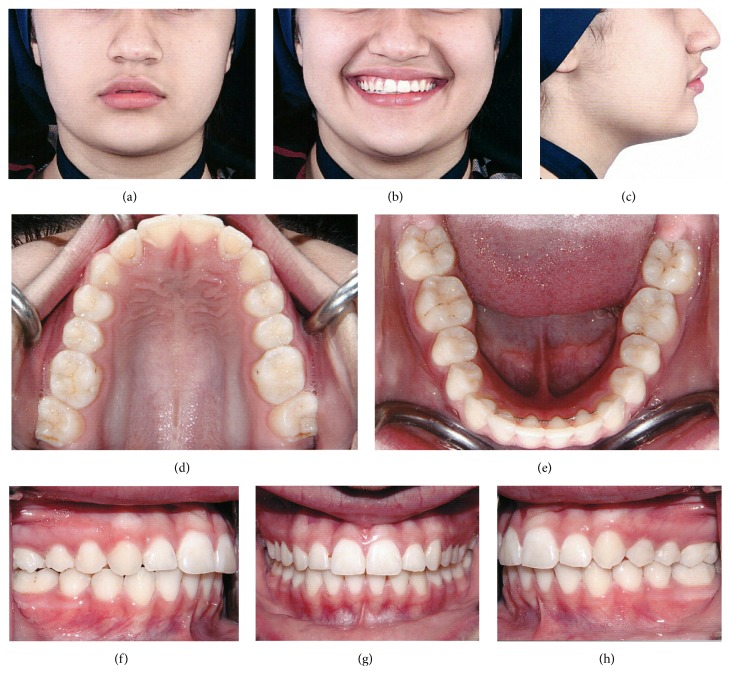
Posttreatment photos: frontal (a); smile frontal (b); profile (c); intraoral views: upper occlusal (d), lower occlusal (e), right (f), frontal (g), and left (h).

**Figure 8:**
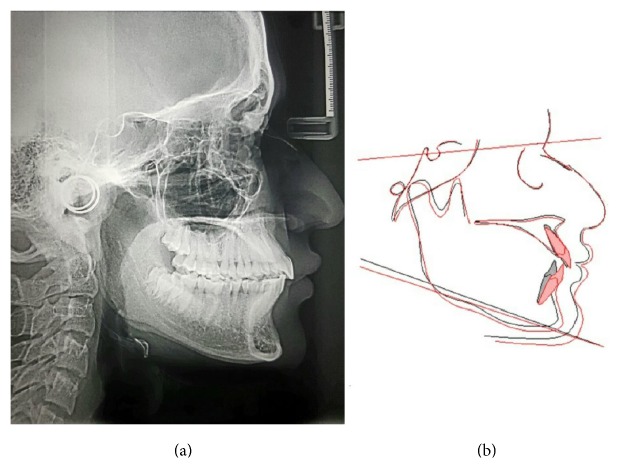
Posttreatment cephalographic image (a); overall superimposition of pretreatment (black color) and posttreatment (red color) cephalometric radiographs (b).

Having finished phase III treatment objectives were met: profile of the patient improved. Crowding was relieved and Cl I relationship with desirable overjet and overbite was achieved (Figure 7). The skeletal effects and increasing of mandibular length were evident, despite the fact that some of the corrections, mainly proclination of lower incisors, were due to dental effects of Twin Block (Figure 8). Overall superimposition of the lateral cephalometric radiographs is also illustrated in Figure 8. Cephalometric measures are shown in Table 2.

## 3. Discussion

Treatment with functional appliances has several well-established advantages. In this case functional appliance treatment caused reducing overjet, patient's profile improvement, and taking care of jaw discrepancies. What is more, the patient's confidence improved and the risk of trauma to the upper incisors was minimized [1].

The selection of functional appliances is dependent upon several factors which can be categorized into the patient factors, for example, age and compliance, and clinical factors, for example, preference/familiarity and laboratory facilities [14]. Twin Block functional appliance has several advantages including the fact that it is well tolerated by patients [15], being robust, easy to repair, suitable to use in permanent and mixed dentition, and compatible with fixed orthodontic appliances, and in comparison with Herbst appliance it has less dental effect; nevertheless, there are potential disadvantages as well as the proclination of lower incisors and development of posterior open bites [11] which in this case was not a big problem because of the normal inclination of lower incisors and the use of fixed orthodontic appliances that can finally eliminate the posterior open bite.

With respect to all the merits mentioned and the writer's experience with the device, the Twin Block appliance was selected.

It seems that age was the only factor which was unfavorable for the functional treatment. Both chronologic age and developmental stages indicated that growth spurt had come to an end. In most literatures [4–11] menarche is assumed to be the last stage of growth spurt. The cervical vertebral maturation stage also pointed to the same founding (CVMS V). Even though we consulted the patient about the disadvantages and limitations, she was still highly motivated to try the treatment with hope of improving her profile.

In a functional treatment, a symmetrical overjet and possibility of advancing mandible without any interference is a critical issue [11]. In our case, the interferences in lateral incisors were a limitation, for which there were multiple protocols. In our case using *z* spring for correction was not a good solution because of remarkable space deficiency and the need for significant amount of striping. Striping however was not a desirable treatment because of possibility of future extraction.

The treatment applied included fixed orthodontic appliances for maxillary arch in order to align maxillary teeth. This treatment has several advantages such as relieving crowding more efficiently, creating a symmetric overjet, and improving the patient's self-esteem. Nevertheless, this treatment takes more time to start Twin Block therapy and causes overjet and U1-SN angle to increase, which were taking care of by functional treatment later.

At the end of the treatment, patient's profile was noticeably improved with favorable treatment effects in cephalometric analysis. SNB was increased by 1.2° while SNA and ANB had reduced by 1° and 1.2°, respectively. Mandibular length also undergone an increase of 2.7 mm. Dental relations for canines and molars were Angle Class I after treatment and overjet was normal. Patient was satisfied with the results with her self-esteem being significantly improved.

Retention was maintained with Hawley appliances for maxillary arch and fixed spiral wire for anterior mandible teeth. Arrangement was then made to visit the patient regularly during the retention phase of treatment. The patient was instructed to wear the retainers for as long as required to ensure stability [16].

Furthermore, she was referred to her general dental practitioner for regular check-ups.

## 4. Conclusions

Functional therapy is an efficient treatment to treat dentoskeletal Class II malocclusions. Twin Block is a functional appliance with several advantages including the fact that it is well tolerated by patients and has great clinical outcomes. Even though age is a limitation, in a patient with good corporation it can create a desirable outcome.

## Figures and Tables

**Table 1:** Normal and pretreatment cephalometric values [12].

Variable	Normal	Pretreatment
SNA	82° ± 3	89°
SNB	79° ± 3	84.8°
ANB	3° ± 1	4.2°
Upper incisor to SN (U1-SN)	102° ± 6	112.7°
Lower incisor to mandibular plane (IMPA)	92° ± 5	91.9°
Interincisal angle	130° ± 10	130.5°
SN-mandibular plane angle	32° ± 4	26°
Face height ratio	65%	75%

**Table 2:** Pretreatment and posttreatment cephalometric values.

Variable	Pretreatment	Posttreatment
SNA	89°	88°
SNB	84.8°	86°
ANB	4.2°	2°
U1-SN	112.7°	114.2°
IMPA	91.9°	100°
Interincisal angle	130.5°	121.1°
SN-mandibular plane angle	26°	25°
Face height ratio	75%	73%
Mandibular length	120.3 mm	123 mm
